# *Candida* Esophagitis in an Immunocompetent Pregnant Woman

**DOI:** 10.1155/S106474499300033X

**Published:** 1993

**Authors:** Jeffrey S. Greenspoon, Seth Kivnick

**Affiliations:** 1 Division of Maternal-Fetal Medicine Department of Obstetrics-Gynecology Cedars-Sinai Medical Center Los Angeles CA USA; 2 Department of Obstetrics-Gynecology Kdiser Foundation Hospital-West Los Angeles Los Angeles CA USA; 3 Room 1738 OB/GYN Cedars-Sinai Medical Center 8700 Beverly Boulevard Los Angeles CA 90048 USA

## Abstract

*Background:* Nausea and vomiting are common during the first half of pregnancy and usually require only supportive measures. When symptoms are progressive and weight loss occurs, treatable causes should be sought by means of upper gastrointestinal endoscopy. We report a case of an immunocompetent gravida with invasive *Candida albicans* esophagitis.

*Case:* The immunocompetent primigravida developed progressive nausea, vomiting, epigastric pain, and a 4.1 kg weight loss during the second trimester of pregnancy. Treatment with metoclopramide and cimetidine for presumed gastroesophageal reflux was not effective. The patient had normal T-cell CD4 and CD8 subsets and was human immunodeficiency virus (HIV) antibody negative. Upper gastrointestinal endoscopy revealed *C. albicans* esophagitis which was treated with oral nystatin. The esophagitis had resolved completely when reassessed postpartum. The use of histamine_2_ blockers is associated with an increased risk for fungal esophagitis and may have been a contributing cause in this case.

*Conclusion:* Pregnant patients with persistent nausea, vomiting, and weight loss should be evaluated by endoscopy for fungal esophagitis.


Infectious Diseases in Obstetrics and Gynecology 1:149–152 (1993)

© 1993 Wiley-Liss, Inc.

### *Candida* Esophagitis in an Immunocompetent Pregnant Woman

**Jeffrey S. Greenspoon and Seth Kivnick**

*Division of Maternal-Fetal Medicine, Department of Obstetrics-Gynecology, Cedars-Sinai Medical Center (J.S.G.), and Department of Obstetrics-Gynecology, Kaiser Foundation Hospital-West Los Angeles (S.K.), Los Angeles, CA*

Abstract

***Background:*** **Nausea and vomiting are common during the first half of pregnancy and usually require only supportive measures. When symptoms are progressive and weight loss occurs, treatable causes should be sought by means of upper gastrointestinal endoscopy. We report a case of an immunocompetent gravida with invasive *Candida albicans* esophagitis.**

***Case:*** **The immunocompetent primigravida developed progressive nausea, vomiting, epigastric pain, and a 4.1 kg weight loss during the second trimester of pregnancy. Treatment with metoclopramide and cimetidine for presumed gastroesophageal reflux was not effective. The patient had normal T-cell CD4 and CD8 subsets and was human immunodeficiency virus (HIV) antibody negative. Upper gastrointestinal endoscopy revealed *C. albicans* esophagitis which was treated with oral nystatin. The esophagitis had resolved completely when reassessed postpartum. The use of histamine$_2$ blockers is associated with an increased risk for fungal esophagitis and may have been a contributing cause in this case.**

***Conclusion:*** **Pregnant patients with persistent nausea, vomiting, and weight loss should be evaluated by endoscopy for fungal esophagitis.** © 1993 Wiley-Liss, Inc.

Key words

*Candida albicans* esophagitis, hyperemesis gravidarum, pregnancy complications, gastrointestinal

We report a case of *Candida albicans* esophagitis in an immunocompetent gravida. We are unaware of other reports of this infection complicating pregnancy. We suggest that *Canadida* esophagitis be included in the differential diagnosis of refractory nausea, vomiting, and weight loss during pregnancy.

### CASE REPORT

A 37-year-old primigravida woman with a history of leiomyomata presented for prenatal care at 8 weeks gestation with a uterus that was 20 weeks size. At the age of 31 years, she had a positive tuberculin skin test and a normal chest radiograph. She refused isoniazid prophylaxis.

Physical examination at that time showed a healthy, thin, black woman with a weight of 58.6 kg and a height of 167.5 cm. Pelvic ultrasonography showed a single, viable, 9.5-week fetus in a gestational sac near the cervix and a large fundal leiomyoma with dimensions of 17 × 10 × 13.5 cm. All routine prenatal laboratory values were normal. The patient declined human immunodeficiency virus (HIV) antibody screening. An amniocentesis done at 15 weeks showed a normal karyotype, 46 XX, and a normal alpha feto-protein.

In the second trimester, she developed increasing uterine pain, tenderness, and loss of appetite. By 17 weeks gestation, her weight had decreased 1.8 kg to 56.8 kg.

**Address correspondence/reprint requests to Dr. Jeffrey S. Greenspoon, Room 1738, OB/GYN, Cedars-Sinai Medical Center, 8700 Beverly Boulevard, Los Angeles, CA 90048.**

Received June 8, 1993
Accepted October 19, 1993

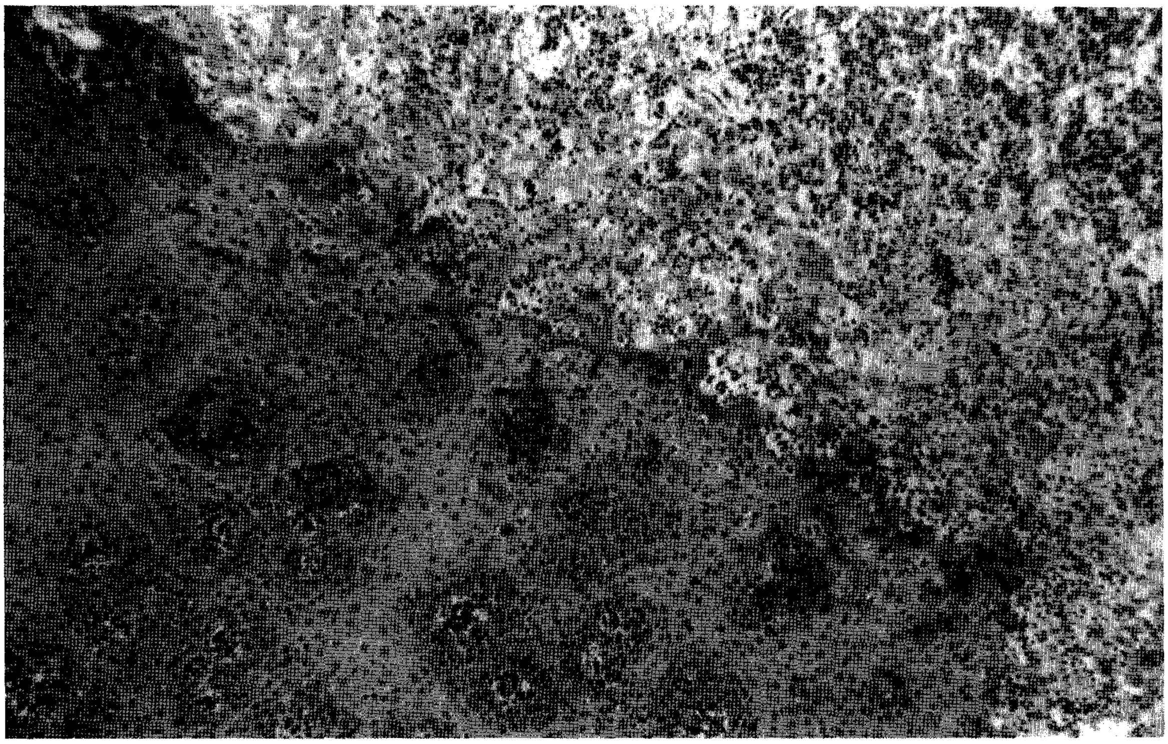

**Fig. 1. Histologic section of esophagus demonstrating invasive *Candida* infection. Acute inflammation and necrotic material are present in the lamina propria. Hematoxylin and eosin stain. ×40.**

Despite enteral supplementation, her weight decreased to 54.5 kg by 22 weeks gestation. She had nausea, vomiting, and a sensation of obstruction in her throat, but denied odynophagia. The uterus was markedly tender and was 30 cm at 22 weeks gestation. Degeneration of the leiomyomata was diagnosed.

Hemoglobin was 10.7 g/dl, white blood cell (WBC) count 8,800/mm$^3$ with a normal differential, platelets 613,000/mm$^3$, total protein 5.6 g/dl, and albumin 2.6 g/dl. All blood chemistries and liver function tests were normal. Ultrasound showed no hepatobiliary abnormalities and confirmed appropriate fetal growth.

Peripheral hyperalimentation was administered. Gastroesophageal reflux was diagnosed and cimetidine, 400 mg b.i.d., and metoclopramide, 10 mg 30–45 min before meals and at bedtime, were prescribed. Her symptoms resolved temporarily; she gained 2 kg. She refused further parenteral nutrition.

At 27 weeks gestation, nausea, vomiting, and weight loss recurred. She had no oral thrush or vaginal candidiasis. She denied having odynophagia, retrosternal pain, hematemesis, or fever. The uterine fundal height had increased to 38 cm. The 1-hr post-50 g glucola blood glucose was 151 mg/dl. A 3-hr glucose tolerance test met criteria for the diagnosis of gestational diabetes (fasting 77 mg/dl, 1-hr 194, 2-hr 167, and 3-hr 90). In order to provide adequate calories, we placed no restrictions and continued the Ensure (Ross Laboratories, Columbus, OH). Subsequent fasting and postprandial serum glucose concentrations remained normal. A 10 French-feeding tube was inserted for enteral hyperalimentation. The tube became kinked and stopped functioning after 1 week. The patient refused reinsertion of the tube until 32 weeks gestation. Upper gastrointestinal endoscopy (for placement of a second feeding tube) revealed the classic patchy white exudates of *Candida* esophagitis. No feeding tube was placed. Fungal culture and histologic section confirmed invasive *C. albicans* infection (Figs. 1, 2). Treatment was initiated with nystatin suspension, 500,000 units swish and swallow q.i.d. Metoclopramide and cimetidine were discontinued.

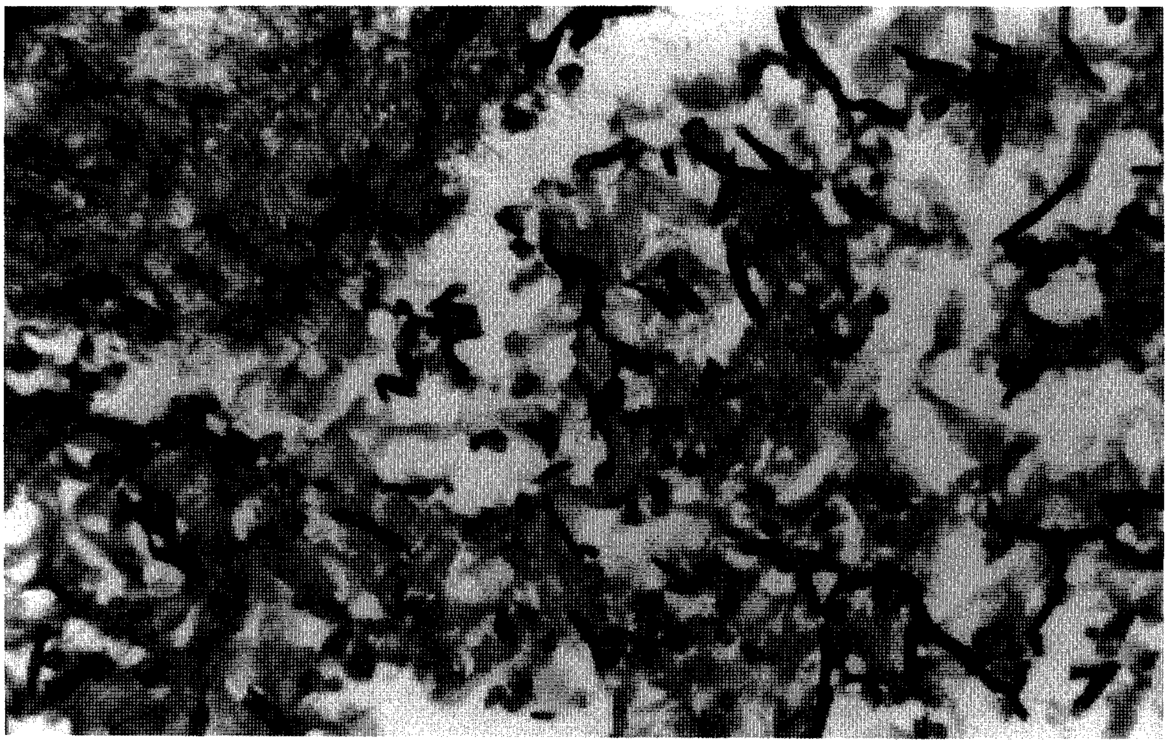

**Fig. 2. High-power view of Figure 1. Histologic section of esophagus demonstrating invasive *Candida* infection. Pseudohyphae are present within necrotic debris. Silver methenamine stain. ×450.**

The patient's and her spouse's serum HIV antibody enzyme-linked immunosorbent assay (ELISA) tests were negative. The complete blood count (CBC), T-cell panel, and serum protein electrophoresis were normal. The WBC count was 10,600/mm$^3$; lymphocytes were 18% of total (normal 15–40%); T-helper cells (CD4) were 48% (normal 32–56%); and T-suppressor cells (CD8) were 24% (normal 17–40%). Skin testing for mumps showed 30 × 15 mm of erythema at 48 hr after injection; coccidioidin caused no reaction. Chest radiograph was normal. During the course of antifungal therapy, the patient's dysphagia decreased, but her oral intake remained poor and abdominal pain increased. She remained euglycemic. After fetal maturity was confirmed, a cesarean delivery was performed at 36.5 weeks for breech presentation and increasing abdominal pain.

The female infant weighed 2,848 g and had Apgar scores of 9 and 9 at 1 and 5 min, respectively. There was a single fundal leiomyoma which extended to the left hemidiaphragm. There was no evidence of *Candida* esophagitis when upper gastrointestinal endoscopy was performed 3 days postpartum.

Five months postpartum, a myomectomy was performed. The leiomyoma weighed 852 g and measured 15 × 15 × 7 cm. Pathologic examination revealed a hyalinized and infarcted leiomyoma. A preoperative HIV antibody test remained negative.

### DISCUSSION

We were unable to find other reports of *Candida* esophagitis during pregnancy in immunocompetent or immunocompromised patients. This diagnosis should be considered in patients with refractory nausea, vomiting, and weight loss during pregnancy. This patient did not have the common symptom of odynophagia or the presence of oral thrush.[1] The nystatin therapy improved the dysphagia, and a repeat endoscopic examination confirmed resolution of the esophagitis. The patient's dysphagia and the histologically proven *Candida*

esophagitis cannot be attributed to the mass effect of the gravid uterus and leiomyoma, although these may have caused some of her epigastric discomfort.

Histamine$_2$ blockers are widely used and are usually well tolerated.[2] A significant association exists between treatment with histamine$_2$ blockers and the presence of fungal esophagitis.[3] The prevalence of fungal esophageal infection was 12 of 72 (16.7%) among patients exposed to histamine$_2$ blockers, whereas only 3.5% of patients unexposed to the drug had fungal esophageal infection. The association is somewhat unexpected because cimetidine has been shown to enhance cell-mediated immunity in humans. Cimetidine augmented delayed hypersensitivity responses to skin tests including the *Candida* antigen in humans with duodenal-ulcer disease.[4] In 4 adult patients with chronic mucocutaneous candidiasis, cimetidine stimulated the immune response to *Candida* antigen and the production of leukocyte migration inhibitory factor, although lymphocyte transformation was not affected.[5] Cimetidine and ranitidine are FDA category B drugs; their use during pregnancy should be limited to instances in which the benefit justifies the risk.

Type I diabetes mellitus has been associated with *Candida* esophagitis. Although this patient met criteria for gestational diabetes mellitus, because she remained euglycemic, we do not think that her glucose intolerance was severe enough to be a cause for the fungal esophagitis. Perhaps the combination of pregnancy, diabetes mellitus, and treatment with a histamine$_2$ blocker may increase the risk for *Candida* esophagitis.

Immunocompetent pregnant patients who have not responded to routine therapy for gastroesophageal reflux should undergo upper gastrointestinal endoscopy to diagnose *Candida* esophagitis. If they have been treated with histamine$_2$ blockers, they are at increased risk for esophageal fungal infection.

In immunocompromised pregnant patients, upper gastrointestinal endoscopy is particularly important to identify opportunistic esophageal infections. As HIV infection becomes more common in obstetrical patients, *Candida* esophagitis and other opportunistic infections will be encountered more frequently. At this time, *Candida* esophagitis is the acquired immunodeficiency syndrome (AIDS)-indicator disease for 15% of HIV-infected adolescents and adults.

Although this patient responded to oral nystatin therapy, immunocompromised patients frequently need prolonged systemic therapy with fluconazole, ketoconazole, or amphotericin B.[6] The safety of these systemic drugs during pregnancy has not been established.

### ACKNOWLEDGMENTS

We thank Samuel H. Pepkowitz, M.D., for assistance in preparing the photomicrographs.

### REFERENCES

1. Haulk AA, Sugar AM: *Candida* esophagitis. In Stollerman GH (ed): Advances in Internal Medicine. Vol. 36. St. Louis: Mosby Year Book, pp 307–318, 1991.
2. Lispy RJ, Fennerty B, Fagan TC: Clinical review of histamine$_2$ receptor antagonists. Arch Intern Med 150:745–751, 1990.
3. Vermeersch B, Rysselaere M, Dekeyser K, Rasquin K, De Vos M, Elewaut A, Barbier F: Fungal colonization of the esophagus. Am J Gastroenterol 84:1079–1083, 1989.
4. Avella J, Madsen JE, Binder HJ, Askenase PW: Effect of histamine H2-receptor antagonists on delayed hypersensitivity. Lancet 1(8065):624–626, 1978.
5. Jorizzo JL, Sams WM Jr, Jegasothy BV, Olansky AJ: Cimetidine as an immunomodulator: Chronic mucocutaneous candidiasis as a model. Ann Intern Med 92:192–195, 1980.
6. Terrell CL, Hughes CE: Antifungal agents used for deep-seated mycotic infections. Mayo Clin Proc 67:69–91, 1992.
